# Genes2WordCloud: a quick way to identify biological themes from gene lists and free text

**DOI:** 10.1186/1751-0473-6-15

**Published:** 2011-10-13

**Authors:** Caroline Baroukh, Sherry L Jenkins, Ruth Dannenfelser, Avi Ma'ayan

**Affiliations:** 1 Department of Pharmacology and Systems Therapeutics, Systems Biology Center New York (SBCNY), Mount Sinai School of Medicine, 1425 Madison Avenue, New York, NY, 10029, USA

**Keywords:** Word Cloud, Tag Cloud, Text Mining, Gene List Analysis

## Abstract

**Background:**

Word-clouds recently emerged on the web as a solution for quickly summarizing text by maximizing the display of most relevant terms about a specific topic in the minimum amount of space. As biologists are faced with the daunting amount of new research data commonly presented in textual formats, word-clouds can be used to summarize and represent biological and/or biomedical content for various applications.

**Results:**

Genes2WordCloud is a web application that enables users to quickly identify biological themes from gene lists and research relevant text by constructing and displaying word-clouds. It provides users with several different options and ideas for the sources that can be used to generate a word-cloud. Different options for rendering and coloring the word-clouds give users the flexibility to quickly generate customized word-clouds of their choice.

**Methods:**

Genes2WordCloud is a word-cloud generator and a word-cloud viewer that is based on WordCram implemented using Java, Processing, AJAX, mySQL, and PHP. Text is fetched from several sources and then processed to extract the most relevant terms with their computed weights based on word frequencies. Genes2WordCloud is freely available for use online; it is open source software and is available for installation on any web-site along with supporting documentation at http://www.maayanlab.net/G2W.

**Conclusions:**

Genes2WordCloud provides a useful way to summarize and visualize large amounts of textual biological data or to find biological themes from several different sources. The open source availability of the software enables users to implement customized word-clouds on their own web-sites and desktop applications.

## Background

Information overload in biomedical research can benefit from methods that can quickly summarize knowledge about specific topics from large bodies of text or data. Word-clouds or tag-clouds are compact visual displays of words where the size and orientation of words represent the underlying importance. Word-clouds can be used to visually summarize information about a specific topic condensing most important terms into minimum amount of space. Word-clouds have been used in other contexts to accomplish this task in many web applications such as summarizing news articles [1]. However, their application in Bioinformatics and Biomedicine has been limited. Desai et al. [2] discussed the use of word-clouds as an alternative way to visualize genes annotations, whereas Oesper et al. [3] developed a Cytoscape Plug-in that summarizes information from a network into a tag-cloud. This Cytoscape plug-in can only accept input from a selected set of nodes and their descriptions, while generating non-interactive and colorless tag-clouds. Although useful, many other possibilities for more broad applications in Bioinformatics and Biomedicine are possible. For example, LigerCat is a web-based system that generates simple looking tag-clouds from MeSH terms of journals, PubMed searches and FASTA sequences [4]. The tag-clouds generated by the Cytoscape plug-in or by LigerCat do not optimize the compactness of the words and use a single font and color. More sophisticated and aesthetically pleasing word-cloud displays such as Wordle exist. There are currently two main web-based applications to create aesthetically pleasing colorful word-clouds from weighted lists of keywords: Wordle, developed by Jonathan Feinberg and indirectly IBM, and WordCram developed by Dan Bernier. Wordle cannot be used outside the web application since its source code is protected, whereas WordCram is an open-source Java library using the Java libraries of the programming language "Processing" to create word-clouds. Processing is a scripting language to quickly create images, animations and interactive content with Java.

Here we present Genes2WordCloud, an open source web application and Java Applet that enables users to create biologically-relevant content word-clouds from several different sources: a single gene or a list of genes, free text, text extracted from a URL of a website, text extracted from abstracts associated with an author, text extracted from abstracts returned from any PubMed search, and word-clouds created from the abstracts of the most viewed articles on BMC Bioinformatics to examine current trends in the field of Bioinformatics.

## Methods

**Figure 1:**
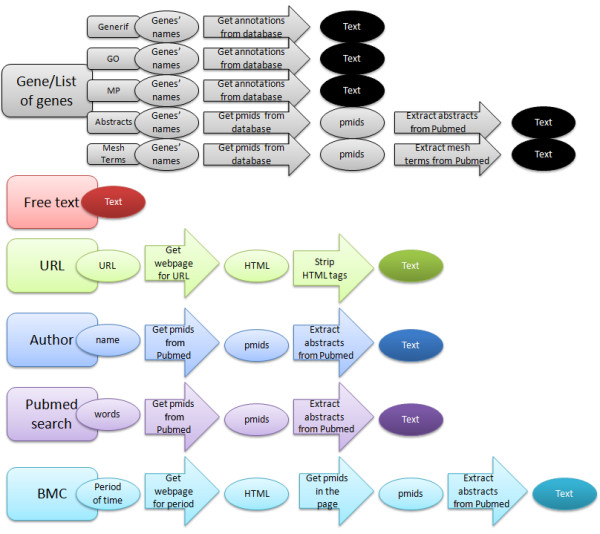
**Fetching text for Genes2WordCloud**. Text to display the word-clouds can originate from six sources. In some cases several steps are taken to convert the input selection to a body of text for further processing.

**Figure 2:**
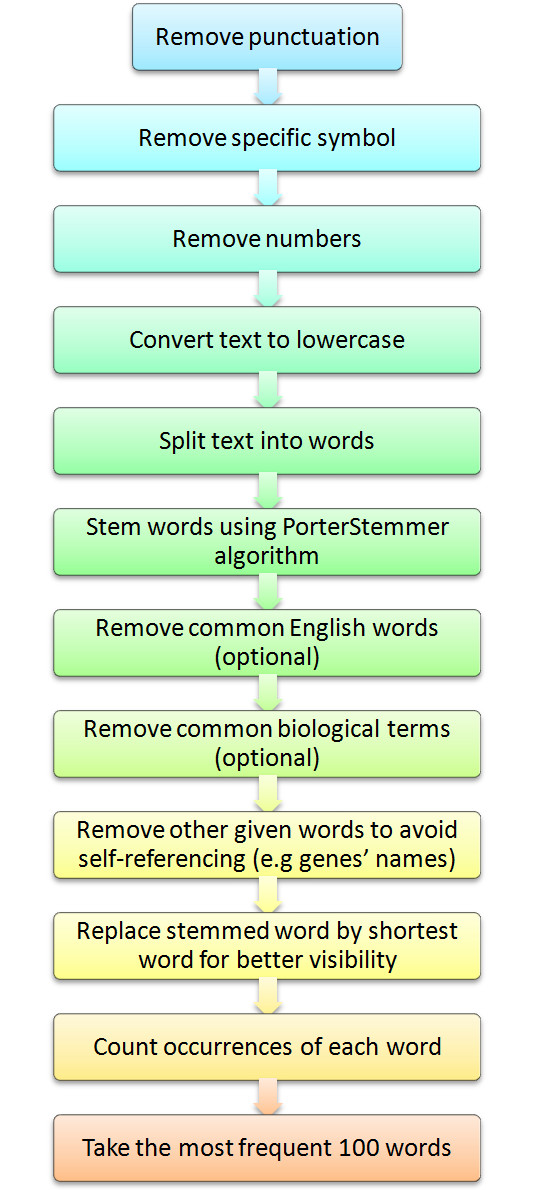
**Text processing pipeline**. The extracted text from the different options shown in Figure 1 is then processed by standard text mining algorithms. Several steps are taken to process the text for word-cloud display.

There are two main steps in creating word-clouds: a) generating the keywords to display, and b) displaying the keywords. The keywords in Genes2WordCloud are generated in several ways depending on the source chosen. In each case the process can be divided into two main tasks: a) obtaining the text related to the user input (Figure 1), and b) text-mining the text (Figure 2). The text for generating word-clouds can be supplied for six different purposes (Figure 1):

a) Obtaining information about a single gene or a set of genes.

The text for a single gene, or a list of genes, is extracted from several alternative sources: GeneRIF, Gene Ontology [5], PubMed abstracts, PubMed MeSH terms or mammalian phenotype annotations from the Mouse Genome Informatics-Mouse Phenotype browser (MGI-MP) [6]. Each of these sources provides text that describes properties of genes. Given a gene ID/s, the software extracts text about the gene/s from these sources.

b-c) Generating a word-cloud from a body of free text or from a give URL.

Free text or text extracted from a URL can also be used to generate word-clouds.

d-e) Generating a word-cloud from articles published by a specific author based on an author's name or from any PubMed search.

Based on an author's name, a word-cloud is created from PubMed abstracts returned for the author, or from any other PubMed search query terms.

f) A word-cloud created from the most popular articles published in the journal BMC Bioinformatics.

All BMC and PLoS journals, including the journal BMC Bioinformatics, provide an updated list of the most viewed articles from a specific journal. Genes2WordCloud provides an option to generate word-clouds from a collection of the most popular abstracts of the journal BMC Bioinformatics.

The different options to obtain text for generating word-clouds are limited to a maximum of 150 abstracts or 500 annotations picked randomly when the queries return more than these limits. Once bodies of text have been extracted from these alternative sources, the text is processed in several steps (Figure 2).

The Porter stemming algorithm is used to reduce words such as "stem", "stems", "stemming" to a single root, e.g., "stem". The identified root is not always a real English word. Therefore, to obtain readable word-clouds, after the stemming of all the words, each stemmed-word is replaced by the shortest word of its family. In addition, some words are completely removed from the text. First, all common English words such as: "the", "is", or "are", are removed. Then common biological terms such as: "experiments", "abstracts", "contributes" are removed. These terms were chosen by hand curation after experimenting with many word-clouds, and users can continually refine this selection by suggesting words to be removed. Text-mining of GeneRIF, Gene Ontology annotations and MGI-MP annotations were also processed to remove common terms. Finally, other terms such as the input gene names, the names of authors, or the keywords from PubMed searches, are removed to avoid self-referencing. Next, words are counted: their normalized occurrence provides their weight used by the WordCram Applet to determine their size, position and angle in the outputted word-cloud. In principle, WordCram starts drawing words in the center of the display while gradually filling the space with other words to maximize compactness. The default angles are horizontal and vertical starting at the center but options for wave, swirl, starting from the left, and few other alternatives are available for locating words. In addition, heaped, mostly horizontal, and random angles are choices available for alternative word orientations. Once the text have been extracted and processed, it is displayed as a word-cloud. Genes2WordCloud uses a word-cloud viewer that is based on the open source Java package WordCram. Genes2WordCloud is implemented using Java, Processing, AJAX, mySQL, and PHP.

## Results and Discussion

**Figure 3:**
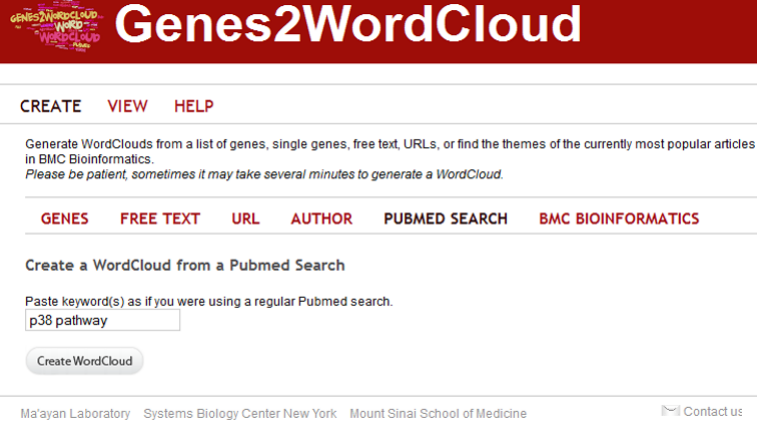
**The Genes2WordCloud user interface**. The initial user interface provides users several options to create word-clouds from different sources: Genes- can be used to create word-clouds from list of genes or single genes; Free Text- can be used to create word-clouds from any body of free text; URL- word-clouds from any given URL; Author- word-clouds for specific authors based on a PubMed query; PubMed Search- word-clouds from any PubMed search; BMC Bioinformatics- word-clouds from the abstracts of the most popular papers published in BMC Bioinformatics.

**Figure 4:**
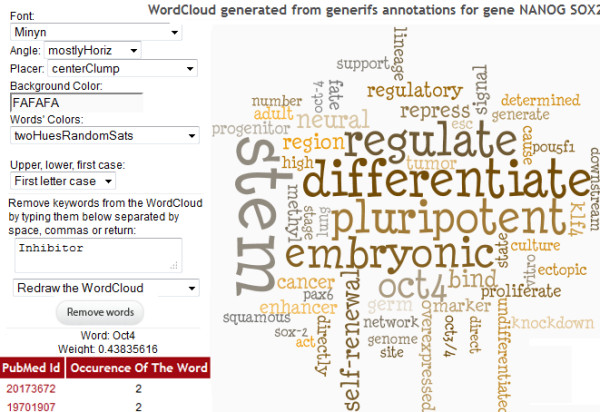
**Word-cloud created using two genes**. Visualization of a word-cloud for Nanog and Sox2 using the Genes option, showing user options to edit the output display.

**Figure 5:**
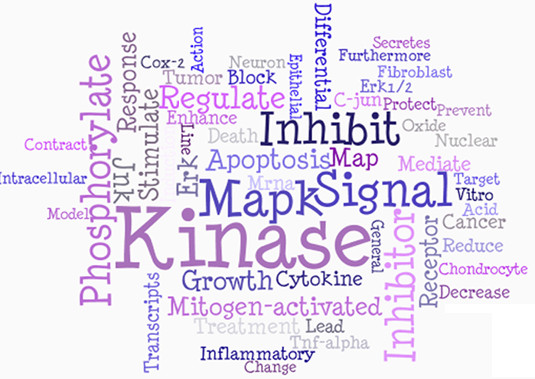
**Word-cloud created from a PubMed search**. A word-cloud created for the p38 pathway using the PubMed search option.

The Genes2WordCloud application contains two sections: the initial interface, which allows users to select the type of word-clouds to generate from the different types of sources (Figure 3), and the actual word-cloud display (Figures 4, 5). Once a selection is made, the word-cloud is displayed and the user is provided with options to interact with the word-cloud and change its properties (Figure 4). Users can change the shape, background color, angle, font, font colors, and font case. The user also has the ability to remove unwanted displayed words. In addition, the origin of the words can be shown by clicking on them, i.e., PubMed identifiers, GO identifiers, or MGI-MP identifiers based on the relevant source used to generate the cloud. Genes2WordCloud is also provided as a zipped package enabling developers to embed the application in their own websites. To demonstrate the functionality of Genes2WordCloud we provide two examples: The first is a word-cloud generated for the genes *Nanog *and *Sox2*; both genes encode transcription factors involved in embryonic stem cells self-renewal and pluripotency (Figure 4). The word-cloud automatically identifies and displays relevant terms such as "differentiate", "pluripotent", "self-renewal", and "*Oct4*", a gene that is often associated with *Nanog *and *Sox2*. The second word-cloud was created with the PubMed search: "p38 pathway" (Figure 5). The algorithm recovered terms such as: "kinase", "signal", "MAPK", "phosphorylate", "apoptosis" which are all relevant to the p38 pathway, a signaling pathway involved in response to stress, cell differentiation and apoptosis.

## Conclusions

In summary, Genes2WordCloud is a new tool that demonstrates that word-clouds can be useful in different contexts to help biologists and biomedical researchers summarize text and extract knowledge from masses of articles and high-content results. Many other applications are possible; the open source of the application enables other developers to create similar applications and utilize the code within their systems.

## List of abbreviations

GO: Gene Ontology; MGI-MP: Mammalian Genome Informatics-Mouse Phenotype.

## Competing interests

The authors declare that they have no competing interests.

## Authors' contributions

AM initiated and managed the project as well as wrote the manuscript. SLJ came up with the initial idea. CB implemented and tested the Genes2WordCloud application, database and website. RD maintains the site and updated the software based on the reviewers' comments. All authors read and approved the final manuscript.
